# What Sustains Wars: Will to Fight Versus Military Might

**DOI:** 10.1111/nyas.70113

**Published:** 2025-11-02

**Authors:** Scott Atran

**Affiliations:** ^1^ Artis International and MAGI Saint Michaels Maryland USA; ^2^ Gerald Ford School of Public Policy and Institute for Social Research University of Michigan Ann Arbor Michigan USA; ^3^ Changing Character of War Centre, Pembroke College University of Oxford Oxford UK

**Keywords:** identity fusion, intractable conflicts, realism versus moralism, religion and nationalism, sacred values

## Abstract

This essay explores the contributions of psychosocial factors in sustaining wars and other extreme group conflicts. It uses the Devoted Actor Framework (DAF) to better understand will to fight (WTF) as a quest for ontological significance. Devoted actors are viscerally and inseparably fused to one another and to their group by moral ideals often held to be sacred and indivisible, and thus highly resistant to negotiation and compromise. The quest for ontological significance involves affirming and securing group identities and associated moral ideals. The importance of WTF and reasons for its postwar neglect are highlighted in an analysis of initial versus later stages of the European theater in WWII. Next is an assessment of the limits of rational realism and the significance of religion and nationalism, followed by an examination of psychosocial factors involved in WTF via behavioral and brain studies in the Middle East, North Africa, Europe, and North America. The discussion section examines the scope and limits of DAF in terms of descriptive and explanatory power and in relation to other approaches to war and group conflict. A final coda addresses the Israel−Palestine conflict and the recent Gaza War to illustrate DAF's relevance for understanding and managing seemingly intractable conflicts.

## Graphical abstract

This essay examines how psychosocial forces shape will to fight through the Devoted Actor Framework (DAF). Devoted actors, bound by sacred, non‐negotiable ideals and fused group identities, pursue a quest for ontological significance that sustains conflict beyond material incentives. Drawing on WWII and recent behavioral and brain research in Middle East conflict zones and beyond, the essay highlights DAF's explanatory power and policy relevance for managing unyielding conflicts.

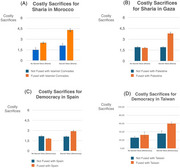

## 1 Introduction: Morality Versus Rationality in War

The Moral Law causes the people to be … undismayed by any danger. [1]

—Sun Tzu, The Art of War (ca 540 bce)

Success in war depends more on moral than on physical qualities. Skill cannot compensate for want of courage, energy and determination. [2]

—British Army Field Service Regulations (1909)

In the fields of political science and international relations, as in foreign policy and military circles, there is an ongoing dispute between “realists” [3] and “moralists” [4] regarding the relative importance of material factors (territory, economy, physical security, balance of power, etc.) versus value‐laden factors (justice, ideals, principles of right and wrong, identity, etc.) in motivating, sustaining, and ending wars. It is a controversy with a very long history.

Consider Leonides, King of Sparta, who arrived at Thermopylae with a small advance guard to hold off a massive Persian assault in 480 BCE. The invading Persian army was thousands strong, and the Greek states had yet to mobilize a response. Plutarch (ca 100 CE) [5] writes that Xerxes, Persia's “King of Kings,” made a written offer he thought Leonides could hardly refuse: “It is possible for you… by ranging yourself on my side, to be the sole ruler of Greece.” Leonides allegedly answered: “If you had any knowledge of the noble things in life, you would refrain from coveting others’ possessions; but for me to die for Greece is better than to be the sole ruler.” Then Xerxes wrote again, “Hand over your arms.” Leonides famously retorted, “Dare come and take them” (μολὼν λαϐέ / molṑn labé). Leonides and his “300 Immortals,” who refused offers to save themselves, were eventually slaughtered; but an inspired Greece would win the war. Or so goes the legend that became part of Western civilization's creation myth.

Herodotus (ca 440 BCE) records that Leonides knew he would die, as did his men when betrayed by Ephialtes, who revealed a secret path around the narrow pass that the Spartans were defending [6]. Leonides dismissed the non‐Spartan Greeks from the battlefield, as they were not bound to the ethos of duty and sacrifice to Spartan honor and glory, which meant never abandoning comrades on the battlefield. In writing about another war shortly afterward, Thucydides (ca 430 BCE), a principal forebear of realism, would argue that such courage and honor are politically instrumental qualities calculated to mobilize men in pursuit of a city‐state's power and greatness [7]. They are strategic assets, not idealized moral virtues or ethical absolutes [8]. From a strategic vantage, Thermopylae could also be considered perfectly rational in delaying action that sacrificed the fighters of one city‐state to preserve the Greek city‐state alliance as a whole. Centuries later, Augustine (ca 420 CE) [9], the forebear of moralism, would not have considered Thermopylae to be an example of a just war in his Christian sense of aiming to restore peace and righteousness; however, he might well have seen it as an earthly display of valor and heartfelt moral virtue (even if not aligned with Christian faith and humility) serving a sense of restorative justice in a defensive war [10].

Although elements of both realism and moralism are recognizable here, Thermopylae actually may be a paradigmatic case of a different theoretical architecture, the Devoted Actor Framework (DAF). Rather than acting purely for material gain or strategic advantage, Leonidas and his warriors were willing to fight and die for non‐negotiable values—honor, freedom, glory, and defense of Hellenic civilization. These values were so deeply internalized that they became sacred, and the warriors’ personal identities fused with the collective identity of Sparta and the broader Greek cause. When bound by sacred values and fused identities, people will resist overwhelming odds and remain impervious to deterrence or exit strategies, however, reasonable or rewarding, even to the point of choosing death over the dishonor of abandoning core values and comrades [11, 12]. Thermopylae exemplifies how sacralized moral commitments can motivate extreme altruism and inspire broader collective resistance. Yet, neither does Thermopylae strictly align with moralist just war traditions. While values of virtue, courage, and moral duty are often viewed in a normative light, as universal ethical standards, the values in play at Thermopylae were cultural‐specific, though absolute, and the altruism exhibited was more parochial than ecumenical.

Throughout history, the most effective combatants, revolutionaries, and insurgents have been devoted actors fused together by dedication to non‐negotiable sacred values like God, country, or liberty. The initiation of war is nearly always planned for maximum force at the beginning to ensure victory. But if defenders resist, or are allowed to recoup, then the advantage often shifts to those with the will to fight (WTF) as they increasingly harness resources against their attackers who are maxed out in terms of what they are able, or willing, to commit: consider Napoleon and then Hitler and their onslaught against Russia, or the US invasions of Iraq and Afghanistan. Across history, those willing to sacrifice for cause and comrades, and for their leaders, have often prevailed against more powerful forces.

Even when defeated and annihilated, the heroism and martyrdom of those with the WTF often become the stuff of legend. Consider the Judeans under Eleazer at Masada, the Alamo defenders under Travis, Bowie, and Crockett [note: that these men supported slavery or other things some deem unworthy is irrelevant to the point here], the Group of Personal Friends who fought to the end defending Chilean President Salvador Allende against Pinochet's putschists. Or take the last holdouts at the Azovstal steel plant in Mariupol, in what might well become a centerpiece of Ukraine's national creation myth along with President Volodymyr Zelensky's celebrated reply to a US offer of evacuation: “I need ammunition, not a ride” [13].

Such legends continue to endure and inspire in political circles, at military colleges, and among the public. And the outcomes of recent and current conflicts continue to demonstrate that nonmaterial factors, such as ideological commitment and collective resolve, can help mobilize forces and yield greater effectiveness on the battlefield. Yet, little sustained or scientific attention is ever paid to understanding why this is so or what to do about it. The European theater in World War II offers some perspective on the issue, where the lessons of the Allies' survival during the war's initial stages have receded under the strategy and success of its later stages.

## 2 WWII European Theater From Dunkirk to The Battle of Moscow (May 1940−January 1942)

From a rational and realist perspective, the expansion of Germany's seemingly unstoppable military power at the start of WWII should have led Britain and the Soviet Union to follow France's example in offering Germany concessions to avoid further bloodshed and total defeat. By late May 1940, British military appraisals were bleak: estimates of the evacuation of British forces from Dunkirk were between 30,000 and 50,000 troops (ultimately, more than 330,000 Allied troops were saved) [14]. The best near‐term hope for Britain was to retrain and resupply 22 of the 80 divisions that had failed to hold the Germans in the first place.

Yet, Britain's War Cabinet decided at the end of May, before the Dunkirk evacuation was complete, not to negotiate with Germany under any circumstances. True, there were those in government, like Lord Halifax, who advocated negotiations with Hitler, and even some Devil's‐Advocate ruminations by Churchill himself to the War Cabinet on May 27. But at day's end, Churchill held true to convictions harbored ever since the Nazis took power, declaring to government officials on May 28: “If this long island story of ours is to end at last, let it end only when each of us lies choking in his own blood upon the ground” [15]. Such Elizabethan sentiments, which harkened back to the existential fight against the Spanish Armada, were further expressed to Parliament in Churchill's most famous war speech delivered later to public acclamation on June 4: “We shall go on to the end…. We shall fight on the beaches… and in the streets, we shall fight in the hills; we shall never surrender.”

The Soviet Union's situation in fall 1941 was, if anything, even more dire. There was no sea or significant navy to impede Germany's surprise invasion in June 1941. Breaking the August 1939 nonaggression pact between Stalin and Hitler, Nazi forces progressed rapidly. US Navy Secretary Frank Knox wrote Roosevelt: “It will take anywhere from six weeks to two months for Hitler to clean up on Russia” [16]. By late November 1941, the Soviets had suffered more than 1 million soldiers dead, over 3 million captured, and loss of most of their coal, iron, steel, and aluminum production [17]. Only 15 tanks remained for Moscow's defense [18].

As the Germans advanced, Stalin called the nation to arms for love of country and duty to Mother Russia rather than to the communist cause. He soon suspended political commissars’ right to overrule battlefield commanders and stopped the campaign to eliminate the Russian Orthodox Church [19]. This strategy somewhat echoed Lincoln's in the Civil War, in which he initially prioritized national unity over abolition despite his belief in a “sacred duty” to make the Union slave‐free [20]. As WWII turned to Soviet advantage, Stalin reimposed commissars on military decision‐making and stressed rule by communist ideals as a principal war aim, much as Lincoln reprioritized emancipation in refusing later Confederate peace offers to rejoin the Union without slavery's abolition. But it was Stalin's Motherland strategy that mobilized popular sentiment and resistance.

Now, one might argue along rationalist and realist lines that Britain and the USSR both ultimately rejected concessions to Germany despite prospects of their likely defeat because of a lack of credible commitment to a deal. And Germany itself arguably went to war with realist aims of recuperating power lost after WWI and expanding into a hegemonic power able to forestall any and all future rivals. But why, unlike France, did Britain and the USSR fight on in 1940−1941 despite military and civilian devastation, economic disaster, and very dim prospects of victory?

One plausible answer is that whereas the deciding elements of Vichy France could accept capitulation and accommodate Nazism and fascism [21], Britain and the Soviet Union had cultural mindsets that would not. Military historian Martin van Creveld argues that France collapsed in 1940 while Britain endured owing to differences in national morale, political coherence, and societal belief in the war effort [22]. The French suffered from a deep‐seated sense of national pessimism and social fragmentation; their political system was discredited, their army demoralized, and their society rife with class tensions and ideological divisions. The memory of the First World War had instilled a pacifist and defeatist culture that left the nation psychologically unprepared for renewed conflict. As political scientist Barry Posen notes, by the interwar period, French nationalism had weakened, especially among the working class and political left, who were alienated from the state and a military that distrusted popular mobilization as a force for unrest rather than resistance [23]. According to Elzabeth Kier, French officers, deeply alienated from the Third Republic, viewed the country's political leadership with suspicion and contempt, leading to weak civilian oversight and further undermining national cohesion [24]. Moreover, the military clung to a rigid, defensive doctrine rooted in the trench‐bound trauma of World War I. It emphasized static defense and strategic passivity, discouraging innovation and offensive tactical thinking.

By contrast, Britain—though militarily weaker—possessed a far stronger will to resist, anchored in a shared national identity, a functioning political system, and widespread consensus on the justness of their cause. Britain's military culture was more flexible and open to reform, and its civil−military relations fostered mutual respect and cooperation. British officers were more attuned to the strategic requirements of modern war and had a stronger commitment to democratic values. This enabled a more adaptive and resilient national response to the Nazi threat. In brief, it was not military inferiority that doomed France but a collapse of will and cohesion, whereas Britain's survival rested on the social unity and moral resolve of its people.

While neorealists like Gideon Rose rightly emphasize France's internal political dysfunction versus Britain's more robust domestic governance and national consensus as factors favoring France's fall and forestalling Britain's collapse [25], perhaps even more telling is what Churchill referred to as “the spirit of the British nation” (radio address, September 11, 1940). For Britain, there was some hope, especially after Dunkirk and the Royal Air Force victory in the Battle of Britain (September 1940), that a German invasion could be prevented, Britain would live to eventually fight again to victory, and more surely so if the United States were to convert its shadow support into direct involvement. Still, Britain's call and apparent will to resist before successful evacuation at Dunkirk, victory in the Battle of Britain, Russia tying down German troops, and US entry into war arguably owed to a deeper “civilizational” commitment to liberal core cultural values and associated traditions and rituals. These fused people to their nation, conveying ontological significance—an inviolable, constitutive, and inextricable sense of “who I am and what we are”—that transcends suffering even unto conquest and death [26].

Hitler offered Britain accommodation on terms more generous than those offered to France, but promised the Soviets only enslavement or annihilation. Yet, unlike France, neither a critical mass of the British nor of the Russians had the psychosocial fiber to abide Nazi domination. Marshal Gregory Zhukov, the top Soviet military leader in WWII, describes the spirit of the Soviet people and its fighters as the embodiment of honor and valor “in the name of the Motherland” (*во имя Родины*) [27]. While Stalin's authoritarian control and socialist propaganda strategically functioned to mobilize the Soviet officer corps and military generally, it was the moral and existential dimension of devotion to Mother Russia and the homeland that galvanized mass resistance, often transcending personal or rational cost−benefit calculations. As Wehrmacht General Günther Blumentritt wrote, like many in Germany, he had overestimated Bolshevism's “fatalistic” effect on Russia [28]:

The behavior of the Russian troops, even in this first battle (for Minsk, June‐July 1941), is strikingly different from the behavior of the Poles and the troops of the Western Allies in the face of defeat. Even when surrounded, exhausted, and deprived of the chance to fight, the Russians never back down. [29]

Indeed, as war went on, British and Soviet notions of fighting spirit were mutually inspiring. Thus, for many Brits, “Soviet soldiers and civilians became a moral gold standard in terms of their dedication to the war effort” [30]. And as Ivan Maisky, the Soviet ambassador to Britain, noted approvingly in his mid‐1940 diary entry: “There is growing [in Britain] a stubborn will, a… determination to fight to the end” [31].

## 3 (Over)Reliance on Material Capacity and Cost Imposition

In March 1941, the United States enacted the Lend Lease Act to supply allied nations at no initial cost with food, oil, and other material resources (and later tanks, ships, and other military ordnance) under the protection of American naval (and later air and land) forces. By the war's end in September 1945, the United States had shipped supplies worth more than 50 billion dollars (nearly one trillion 2025 dollars), principally to Britain and the Soviet Union to help them reconstitute and then ramp up their own forces. Upon American entry into war in December 1941, President Franklin Roosevelt committed the nation to unconditional victory over the Axis powers through the generation and application of overwhelming force, reprising in part the commitment to actions taken by President Lincoln to win the US Civil War.

Roosevelt believed, and soon convinced the public and business community, that its overwhelming economic power—untouched by early military defeats—could be rapidly transformed into military power and eventual victory. This belief was rooted in a strong faith in liberal capitalism, which greatly helped to successfully mobilize the war effort—a faith and capacity underestimated (at the outset at least) by Germany and Japan. Spearheaded by US Army Chief of Staff George Marshall, and in coordination with the business community, by the end of WWII, American military forces increased 50‐fold and the United States accounted for half the world's wartime industrial production [32]. A primary focus on the Allies' superior material capacity and manpower reserves resulted in the destruction of Axis military infrastructure, which did not have the means to reconstitute itself. The political regimes dependent on that infrastructure collapsed, enabling a wholesale transformation of former enemies into friends or clients. From a US government vantage, material superiority also expedited victory in the Cold War insofar as “the arms race [with the United States] helped to create the economic conditions [in the USSR] that preceded collapse” [33].

Nevertheless, a paramount focus on material capacity to the neglect of WTF in subsequent regional wars—Vietnam, Afghanistan, Iraq—has arguably carried woeful costs in lives, treasure, and policy failures [34]. Traditional military and law enforcement doctrines since WWII have continued to rely almost exclusively on use of overwhelming force and deterrence through cost imposition [35]. As one official US Defense Review put it: “In confronting the range of security challenges it will face in the 21st century, the United States must constantly strive to minimize its own costs in terms of lives and treasure, while imposing unsustainable costs on its adversaries” [36]. “Our goal,” stated US Defense Secretary (later Secretary of War) Pete Hegseth, “is to make the costs [of war] too high” [37]. Yet, a strategy to make violence against a society too costly to pursue is unlikely to dissuade devoted actors. For example, suicide bombers fail to respond to utilitarian cost−benefit judgments in their willingness to sacrifice their lives and even their families, that is, the totality of material self‐interests [38].

In testimony to Congress in 2022, Gen. Scott Berrier, US Defense Intelligence Agency director, acknowledged misjudging Ukraine's ability to resist Russia: “I questioned their will to fight. That was a bad assessment,” which risked Ukraine's early defeat [39]. Following the Taliban victory after two decades of war, Gen. Mark Milley, Chairman of the US Joint Chiefs of Staff, blamed “strategic failure” in Afghanistan on neglecting the “intangible” factor in war: “We can count the trucks and guns and the units and all that. But we can't measure a human heart from a machine” [40]. As President Biden noted at the time: “We gave [Afghan forces] every tool they could need…. What we could not provide them was the will to fight” [41]. In 2014, when ISIS routed US‐backed Iraqi government forces despite vastly inferior manpower and no heavy arms or air force, President Obama endorsed the lament of his Director of National Intelligence: “We underestimated the Viet Cong… we underestimated ISIL and overestimated the fighting capability of the Iraqi army… It boils down to predicting will to fight, which is an imponderable” [42, 43].

Despite conceding the importance of WTF *after the fact*, consensus remains that it is “imponderable,” when it is not. Without rigorously assessing nonmaterial sensibilities, including among civilian populations, conflict can appear intractable or only resolvable with massive force; and allies, adversaries, armies, and peoples will continue to be overrated or underrated, with resultant squandering of lives and resources, and greater insecurity overall [44].

## 4 The Limits of Realism and Rationality

Ever since WWII, *Realpolitik*, or political realism, has dominated the international relations field [45], as well as foreign policy and military strategy [46]. Realism holds with von Clausewitz's dictum that: “War is a mere continuation of politics with other means” (“*Der Krieg ist eine bloße Fortsetzung der Politik mit anderen Mitteln*”) [47].

Structural realism proposes a parsimonious theoretical framework to explain behavior within the international system of nation‐states, based on four axioms [48]: (1) territorially organized states are the primary actors in world politics; (2) states behavior is rational (e.g., their preferences are transitive, and they compete in regard to alternative behaviors in terms of diminishing marginal utility); (3) states pursue security and calculate interests as a function of their power relative to others in the international system; and (4) the international system is characterized by anarchy (absence of effective authority to ensure compliance with agreements or norms). Structural realism offers coherent explanations of how wars start, are sustained, and end based on concepts and assumptions related to national material capabilities, actual power, perceived power, major power status, coalition formation for balance of power, as well as national “will” [49]. Here, “will” refers to a nation's resolve to commit its material resources—infrastructures, money, personnel, time, cooperative pooling—to defend itself or go on offense [50].

Beyond these axioms, further assumptions within the structural realist framework produce quite different analyses and predictions of how states behave and consider war. Thus, for “defensive” realists, such as Kenneth Waltz [51], politics and war aim at maximizing security through “balance of power.” For “offensive” realists, like John Mearsheimer [52], the goal is rather power maximization, which proscribes acceptance of any balance of power or status quo. This Hobbesian view of great power conflict owes to the belief that the absence of a reliable and effective enforcement authority in the anarchic international system creates powerful and incessant incentives for states to seek control over the scarce goods on which national life depends (e.g., territory, natural resources, population) at the expense of rivals.

Post‐Cold War challenges to American power from belligerent Islamist revivalism, and a resurgent China, to revanchist Russia, have propelled offensive realism to the forefront. In this view, for example, the Ukraine War seems an inevitable result of US‐led NATO expansion into Russia's former security space when Russia was weakest, where then a reinvigorated Russia became hellbent on reversing America's opportunistic power play. Also, from this perspective, continuing conflict between China and the United States is inevitable owing to the inability of the anarchic international system to enforce any peace between the Great Powers (although a novel threat of near‐certain mutual nuclear annihilation may forestall all‐out war). Graham Allison has dubbed the structural rivalry between established powers and their challengers as “The Thucydides Trap” [53]. Just as Thucydides saw the Peloponnesian War as being unavoidable given the rise of Athens’ relative power at Sparta's expense, so history repeats itself: Great Powers rise while others fall, as today China's relative power increases while that of the United States supposedly declines. [Note: Although Allison grants that such “objective” structural factors may favor war, he takes a more nuanced realist position than Mearsheimer in cautioning that spiraling competition and zero‐sum conflict need not be inevitable because humans exert agency to alter outcomes “through perceptions and emotions and psychology”] [54].

Yet, as James Fearon famously argued [55], whether in an interstate or dyadic context [56], if wars are very costly, then *ceteris paribus*, it is irrational for states to reject negotiated settlements to avoid the costs of fighting. Fearon echoes Thomas Shelling's view of war as a rational “bargaining” process (e.g., over international borders or makeup of national governments) [57]. From this vantage, wars start not because of power lust or anarchy as such, but for lack of information about true intentions and capabilities of rivals or illusions about one's own capabilities [58], and because of unresolved doubts about the ability of states to make binding commitments to any deal. Wars allay these uncertainties, and end when these are credibly reduced or resolved [59].

Bargaining analyses highlight the uncertainties concerning “military capabilities and willingness to fight,” viewed basically in material terms: building weapons, mobilizing troops, pooling resources, and so forth [55]. In addition to uncertainties about material capabilities and national will, states may go to war because of the inability to negotiate concessions over “issue indivisibilities” that will not admit compromise: for example, value‐laden rights (e.g., to practice a religion) or sacred grounds [60]. Even if issues are seldom indivisible “by their very natures,” they still can be “*effectively* indivisible” owing to “domestic political and other mechanisms” that lie outside rational bargaining analyses. Fearon grants that such considerations “based on irrationality” or “pathological domestic politics” can be relevant to explanations of war (p. 409) [55]; however, he claims that their relevance cannot be ascertained without first specifying the “causal mechanisms making for war in the ‘ideal’ case of rational unitary states.” But whereas rational realism's contribution to understanding conflict is undeniable and important, its claim to constant causal primacy in the production and analysis of conflict may itself be more a matter of faith than science.

To summarize: Structural Realist (Defensive, Offensive) and Rational Bargaining theories, which form the dominant paradigms in foreign policy and military circles, focus on the scope and limits of how states, idealized as unitary actors, amass and deploy information and material fighting capacity to pursue and maintain power in competition with other powers. Issues exogenous to such theories, such as domestic politics, substate actors, and third‐party involvement, are usually introduced on a case‐by‐case basis, in an ad hoc rather than structurally principled manner. Least theorized and systematically analyzed, however, is the psychosocial character of WTF despite its critical, and sometimes decisive, role in the pursuance or resolution conflict [44].

## 5 The Quest for Ontological Significance

Based on behavioral and brain studies with frontline fighters, terrorist and militant groups, and support populations [11, 61, 62, 63], it appears that many conflicts—notably seemingly intractable wars that are most difficult to frame settlement terms for—are existential conflicts over mutually exclusive claims to ontological significance. In line with realist and bargaining theories, wars do not end until a new security architecture is achieved that is less threatening to the victor or principal rivals in a conflict. Still, war and settlement may not be primarily about material power and related uncertainties but about ensuring that the group's ontological survival and significance is credibly secured [26].

Ontological significance is an affirmation of group identity—whether a nation, religion, or movement—that viscerally fuses individuals to the group and its values (and sometimes leaders) via costly commitments to core cultural, or transcultural “civilizational,” values often held as sacred and indivisible, and thus highly resistant to bargaining. The idea of ontological significance uses insights from psychological theories of individual “quest for significance” and nation‐states’ “ontological security” from international relations [64]. Quest for significance is a need to have one's social worth affirmed, and “motivates behavior that aims to affirm, realize, and/or show commitment to an important value” [65]. When individuals feel their sense of social worth undermined and their values ignored or violated, they may resort to violent acts to secure outcomes better than the status quo. This complements what sociologist Anthony Giddens has deemed the pursuit of “ontological security”: “confidence or trust that the natural and social worlds areas they appear to be, including the basic existential parameters of self and social identity” [66].

Ontological significance, then, is not primarily about material status or physical survival. Consider China and the United States risking war for Taiwan, or Russia and the United States risking war over Ukraine. Taiwanese or Ukrainian independence poses no realistic security risk to the territorial integrity or independence of the three great powers, whose internationally recognized status has never been more secure [67]. On the contrary, continued conflict only further risks endangering the security of each power. But even if Taiwan and Ukraine were to disarm, conflict would likely persist because material security issues are not the engine of great‐power politics here. Driving policies are issues of respect, honor, and national significance, along with the sentiment on all sides that the core values of democratic liberalism are largely incompatible with autocracy in the competition for global influence.

In reality, states are not unitary actors. Rather, state actions emerge from the interests and beliefs of the people who inhabit and rule states. A nation‐state is an “imagined [though not imaginary] community” of interests, beliefs, and behaviors, along with symbols and artifacts that evoke them [68]. I refer to individuals willing to fight and die to secure ontological significance for their primary reference groups (e.g., family, tribe, nation) as “devoted actors” in contrast to rational actors [11, 20]. Devoted actors tend to be the most effective warfighters or their most ardent supporters [69]. They behave in ways that can violate rationalist assumptions (e.g., transitivity of preferences, action vis‐a‐vis alternative behaviors in terms of diminishing marginal utility) [70]. Revolutionary and insurgent movements are often led and populated by devoted actors. On the battlefield in Iraq, for example, our research group found evidence, both psychological and physical (e.g., casualty rates), that ISIS fighters and those of the secular Marxist‐Leninist PKK were more likely to be devoted actors willing to fight regardless of risks or costs than were regular army or militia [11, 61].

The US military has long harbored notions of a ten‐to‐one force ratio for counterinsurgency because of difficulties in attacking a dispersed enemy, while underplaying commitment to their cause (although undermining insurgents’ “legitimacy” is also emphasized) [71]. Thus, Defense Secretary Robert McNamara informed President Lyndon Johnson that increasing troop levels to 175,000 early in the Vietnam War “was too small to make a significant difference in the traditional 10–1 government‐guerrilla formula”; however, those forces were to suffice as the military situation evolved (but never did) into a conventional war [72]. Indeed, since WWII, revolutionaries and insurgents willing to sacrifice for a cause and comrades have prevailed with 4 to 10 times less resources (manpower firepower, logistics, training, etc.) than state armies and police that rely mainly on material incentives (e.g., pay, promotion, punishment) [73, 74].

Bargaining theory holds that because war is costly, there must be rational incentives to go to war to get a good deal, where diplomacy cannot because of the uncertainty of information and of credible commitment to a settlement. Yet, in cross‐cultural studies, we find that military and diplomatic options link to different notions of “costs” and “benefits”: subjects are willing to allow the deaths of most hostages under a military option, but few or no deaths under a diplomatic option (also exemplified in Israeli policy in the recent Gaza War) [75]. This arguably owes in part to the moral and emotional force of military action, which binds group members in a “covenant of blood” through demonstrations of readiness for costly sacrifice, whether one's own blood or of precious others in the group [70].

In other fields and survey studies, we find that leaders and populations reject peace deals involving sacred values, like holy land or national sovereignty [76, 77], regardless of certainty about the intentions of an adversary or third‐party guarantees. Moreover, offers of monetary or other material payoffs to sweeten deals tend to backfire against rational expectations, increasing support for violence against those deals [12, 78]. Such values and associated objects, places, and events may not be indivisible “by their nature”; however, they are effectively indivisible because they are also *affectively* so, that is, imbued with singular emotional force and ontological significance.

## 6 Religion and Nationalism

The disputes over realism and rational bargaining versus moralism in generating, sustaining, and ending wars cannot simply be reduced to claims about the importance of “ideology” or “culture.” Consider religion and nationalism.

Nationalism and religion are typically viewed as distinct social and political categories. Nationalism, which first emerged as an explicit political ideology and mass movement in Germany and Italy following the American and French revolutions, usually centers on shared language, territory, historical narrative, and sociocultural norms [79]. Religion, at least since the rise of large cooperative societies [80], is generally associated with transcendent moral understandings and associated practices that give ultimate meaning and divine sanction to life and physical experience [81].

Nevertheless, nationalism and religion exhibit structural and functional similarities that make them comparable in affirming the group values and identities involved in the creation of modern societies and sociopolitical movements. Both generate what French sociologist Emile Durkheim deemed “collective consciousness” (*conscience collective*), that is, ideas and beliefs that bind people together into collectivities that are perceived to have lives of their own beyond the present time and individuals belonging to them [82, 83]. Both uphold unification of communities and legitimization of authority, while reducing “transaction costs” in mobilizing people for costly collective efforts, including monumental works and wars [84, 85]. Both deploy powerful rituals that express allegiance through material and emotional sacrifices. These are symbolically iterated in repetitive communal gestures: with banners and songs, parades and pledges, pilgrimages and morality tales, artwork and theatrical displays, and so forth [86, 87]. Such practices embed collective identity, cultural cohesion, and social solidarity in the consciousness of potentially vast numbers of strangers: by integrating, orchestrating, and sanctifying an interpretive framework and civic order for understanding and acting in the world and accepting one's place within it [68, 88].

Realists generally do not consider religion to be a primary driver in international relations, including war. For classical realists, religion may influence domestic politics and color how leaders frame conflict [89], helping them to justify political aims and mobilize popular support [90]. Nevertheless, religion does not override state interests or power politics in the struggle for survival or dominance in an anarchic world.

Although more recent realists also generally downplay religion as a factor in interstate relations, they do sometimes acknowledge religious and cultural traditions that influence state behavior “as potent sources of identity” [91]. This is particularly evident for current conflicts in the Middle East, including among US foreign policymakers influenced by evangelical Christianity [92]. Nevertheless: “Although religion plays a central role in the lives of individuals and communities, it seldom determines how states behave in the international system” [93]. This presumably also applies to states that conceive of their role in the world in religious terms, like the Islamic Republic of Iran and the State of Israel, where religious interests are essentially idiomatic expressions of national interests that prioritize power and security. Transnational terrorist movements, like Al Qaeda and ISIS, may have transcendent ambitions that do not readily reduce to realistic power plays; however, from a realist vantage, such movements are less important in themselves than in how they may affect state behavior or balance of power.

Defensive realists, like Walt, and offensive realists, like Mearsheimer, do acknowledge that religion should be included in studies of international conflict to gain a fuller picture of global interactions, as between Israel, Iran, and the Palestinians. But rather than attempt to deal with possible structural and systemic implications of religion or cultural values and other moral or ideological aspects of international conflict, realists hold that these should be discouraged among decision‐makers as deleterious to the conduct of foreign policy (e.g., the United States promoting religious freedom abroad or Iran promoting revolution as a religious duty). This is because overconcern with such factors obscures the true material interests and power calculations that ultimately determine international relations and how to best manage them. By contrast, constructivist and normative theorists, who focus on moral dimensions and limits of power and motivations for war, put much greater stress on religion and cultural mores, without necessarily denying the importance of material pursuits and power concerns [94].

In contrast to religion, nationalism for some realists is recognized as a systemically important factor in international relations and war—although more as a tool of power politics, state interests, and popular and military mobilization than as a cause in itself that affirms collective identity and worth (e.g., through self‐determination, in righting a historical grievance, performing a sacred duty, etc.). Thus, for Mearsheimer, who sees the “human tragedy” of states inevitably seeking security and survival through aggressive expansion, “Nationalism is the most powerful ideology on the planet” (p. 122) [91]. Nationalism's emotional and cohesive power is critical to forging the identity and reality of the nation state, “the ideal political unit in the modern world… where each nation gets its own state” (p. 94) [91]. Supposedly rooted deeply in the tribal instincts of human nature, nationalism is often tied to shared ethnicity, cultural history, territory, and language. Nationalism thus tends to be exclusionary, generating hostility to outsiders and overriding liberalism, universal values, and concern for panhuman rights. Nationalism incites the passions of a population and channels and limits its action in ways that material interests alone cannot. Indeed, material interests, such as the need or desire to control territory and populations, are often determined by nationalism.

Although both nationalism and religion offer a sense of belonging and render willful cooperation possible among anonymous individuals, they also engender clear demarcation between in‐group versus out‐group boundaries. Religion distinguishes between believers and nonbelievers in terms of moral obligations tied to communal membership, often shunning nonmembers of the religious community. Nationalism similarly separates citizens from noncitizens in terms of social norms and cultural heritage, often excluding those deemed foreign or disloyal. Identity‐affirming rituals, particularly if costly and hard to fake or imitate, only harden boundaries between in‐group and out‐group [95]. Such identity‐forming function becomes particularly salient during crises, as in times of war, when individuals turn to religious or national communities for meaning and solidarity. When national and religious narratives combine, or are threatened, then motivation, justification, and intensity of conflict may ramp up even more: examples include the American Civil War [96], Mexican and Russian Revolutions [97], the Spanish Civil War [98], and the ongoing Israel versus Palestine and Iran conflicts [99].

In brief, nationalism and religion both can provide broad ideological or moral frameworks that convey ontological significance, while enabling states (or subnational and transnational movements) to mobilize their populations and combatants in pursuing power and strategic interests, as some realists and most moralists allow. Yet, even when structurally critical and pervasive in international relations, religion and nationalism do not necessarily produce the psychological fusion, non‐negotiable commitment, and readiness for costly sacrifice that define devoted actors. Nationalism and religion can provide the ideological and moral soil from which devoted actors may emerge, but we shall see that other factors, most notably identity fusion and sacralized values, are the causal roots that produce devoted actors.

## 7 Costly Sacrifices and the WTF

For several years, a research partnership between Artis International, Oxford University's Changing Character of War Centre, Spain's Universidad Nacional de Educación a Distancia (UNED), and the Universitat Autònoma de Barcelona has focused on willingness to fight and other costly sacrifices: from giving up material or social benefits to abandoning family and launching suicide attacks. The conceptual frame is the “Devoted Actor” [11], which focuses on the spiritual dimension of human conflict [61]. Devoted actors are individuals who share cherished and protected values with members of a group with which they are viscerally united. Devoted actors are particularly prone to make extreme and costly sacrifices when personal identities “fuse” with collective identity in a primary reference group [100]—often expressed as a family‐in‐arms [101] of imagined kin (e.g., Motherland, Fatherland) [70]—and in defense of core cultural values that are often held to be sacred [26, 102].

**FIGURE 1:**
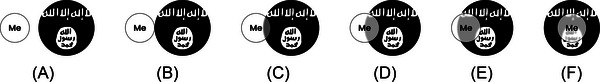
Pictorial measure of identity fusion used with Islamic State fighters.

Identity fusion is a construct that has been assessed by pictorial, verbal, and dynamic alternative measures. For example, in the pictorial version [103], participants are asked to consider a pair of circles where one circle represents “Me” and a larger circle represents the “Group” or the “Value” (tagged with a flag or other identifying icon), with different degrees of overlap between circles (Figure 1). Participants who consider the F option think and behave in ways different from those who choose any other pairing: they wed personal identity (“who I am”) to a unique collective identity or value (“who/what we are”).

In the dynamic version [104], fusion is measured by asking participants to drag a small circle (“Me”) to a position that best affirms their relationship to a large circle representing a Value of a Group. A person is considered “fused” with the value or group when they place themselves in the very center of the large circle. Prior studies, from the battlefields of Libya [101] and Ukraine to America's culture wars [69], indicate that total fusion expresses a visceral and inseparable part of one's identity. Identity fusion resembles social identity theory [105] but emphasizes kin‐like bonding of people and values in ritualized oaths and acts that often involve shared pain and suffering [87], rather than (conceptual and emotional) evaluation of one's self‐image as belonging to one group compared to others.

Fusion is one reliable predictor of willingness to make sacrifices for a group or greater cause. As already indicated, another predictor of self‐sacrifice is when the group cause becomes a sacred value. Whether religious or secular, like God's Commandments or Presumption of Innocence, sacred values are non‐negotiable, regardless of material risks, rewards, costs, or consequences. Although prior work on sacred values examined stated unwillingness for tradeoffs among US student populations [106, 107], the authors of these and similar studies argue that such values are only “pseudo‐sacred” [108] because tradeoffs are possible: for example, to save many lives [109]. In real‐world conflict and battlefield conditions, however, we find actual (and not merely stated) willingness to fight, die, and sacrifice even family and friends for sacred values [110], as with some suicide bombers [111].

Although much more is known about economic decision‐making than value‐driven behavior, features of sacred values that we have validated are: immunity to material tradeoffs; insensitivity to temporal and spatial discounting; resistance to social influence; blindness to exit strategies; privileged link to emotions; distinct neural signatures; and related actions dissociated from calculated risks, costs, or rewards [112]. In our various studies, we measured sacred values by first assessing rejection of monetary incentives, including actual money offers (e.g., in refusing to anonymously write “I don't believe in God” on a piece of paper, then burning the paper and taking the money) [113]; however, in other studies, we cross‐checked monetary refusals against rejection of social benefits (e.g., status awards) and social pressure (from peers, family, community), and use of fusion with value as a proxy for sacred values [12, 69, 114, 115]. Participants in our studies were asked about their willingness to make costly sacrifices for any given group they were fused with or value they held sacred. Different items for costly sacrifice were adapted for the frontline (e.g., kill civilians, undertake a suicide attack, torture women and children) versus with civilians or online (e.g., lose job or income, go to jail, fight), corresponding to different realities that allowed for variation in responses. In all studies, the covariance (Cronbach's alpha) among items of sacrifice was uniformly reliable.

When fusion and sacred values co‐occur, they can interact to produce devoted actors willing to sacrifice all, including their lives and loved ones. To be sure, identity fusion and sacred values are not limited to religious and far‐right or far‐left groups and beliefs, and they may well serve the causes of tolerance and peace, as in the words and actions of Martin Luther King and Mahatma Gandhi. Here, though, I am primarily concerned with how identity fusion, sacred values, and two other relevant concepts, spiritual formidability and trust, can be treated as interacting cognitive variables that generate extreme behaviors in the service of uncompromising religious and political actors.

There is more to this than merely collections of people, their behavior, ideas, and values. There is also the web of relationships that make the group more than the sum of its individual members [116]. It is this networking of members that distributes thoughts and tasks that no one part may completely control or even understand [117]. Case studies of suicide terrorism and related forms of violent extremism suggest that “people almost never kill and die [just] for the Cause, but for each other: for their group, whose cause makes their imagined family of genetic strangers—their brotherhood, fatherland, motherland, homeland” (p. 33) [70]. But here, I leave aside the important study of group dynamics in forming the ties that bind devoted actors.

## 8 Devoted Actor Studies

**FIGURE 2:**
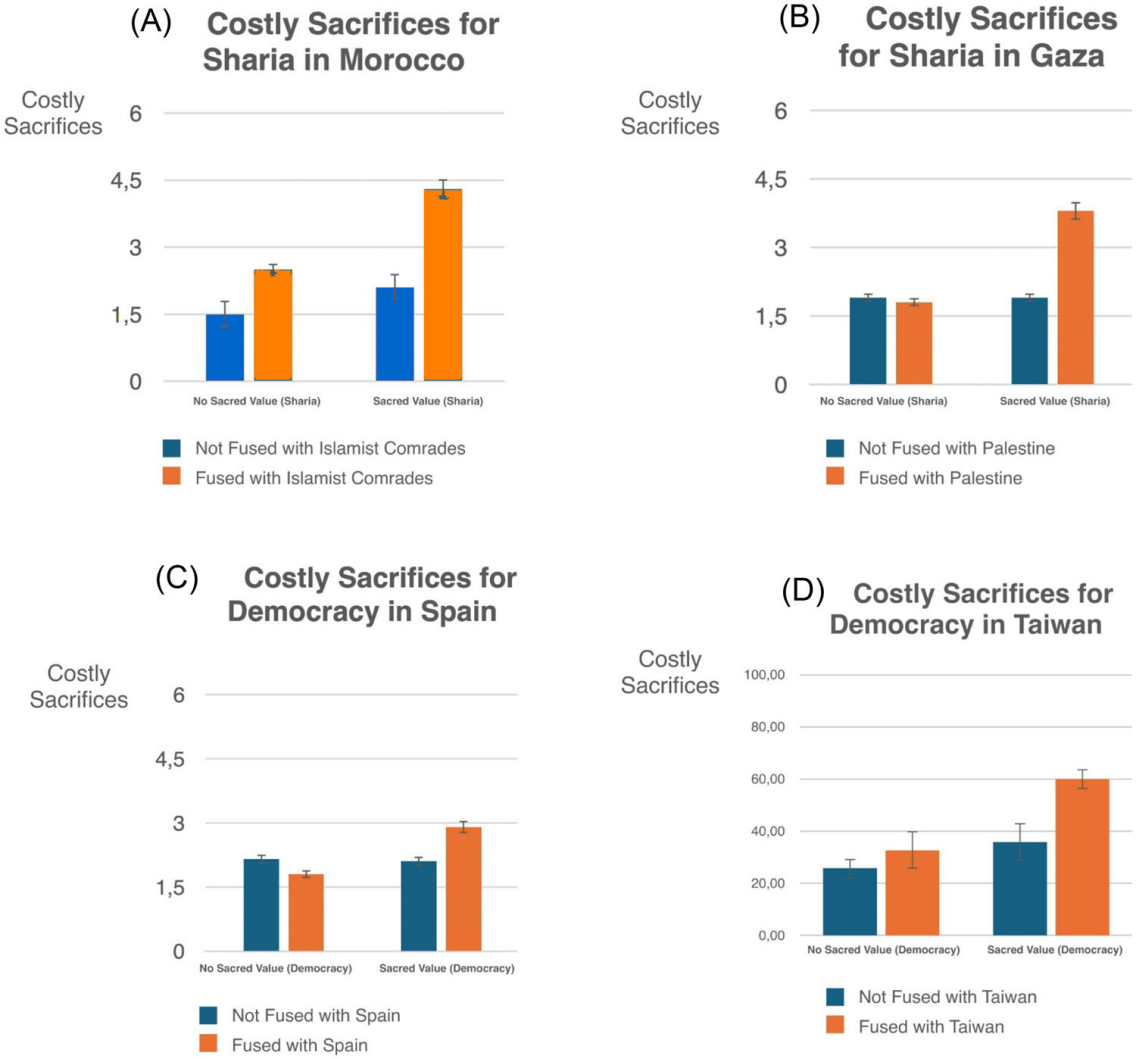
Costly sacrifices are maximized via the interaction of sacred values and identity fusion. (A) In jihadi‐supporting Moroccan neighborhoods in 2014, people who viewed strict imposition of Islamic law, or Sharia, as a sacred value and who identified closely with a kin‐like group (i.e., were fused with the group) expressed the most willingness to sacrifice for Sharia, including fighting and dying. Only those who considered Sharia as a sacred value and were fused with a family‐like group of comrades were above the midpoint (i.e., more willing than not to make costly sacrifices). (B) In 2025, among a representative sample of Gazans after 15 months of war, those who considered Sharia rule over the land to be a sacred value, and whose identities were fused with Palestine, expressed the most willingness to sacrifice for including fighting and dying. Only Gazans who considered Sharia as a sacred value and whose identities were fused with Palestine were above the midpoint in measures of willingness to make costly sacrifices. (C) In 2014, Spaniards fused with Spain reported the highest willingness to make costly sacrifices for democracy as a sacred value, but below midpoint and only when explicitly primed with threat. (D) In 2025, an island‐wide survey of key Taiwanese actors (*N* = 613, mean age = 37, 72% male, SD = 12.3) likely to be engaged by an invasion (military reservists, first responders, community leaders, and communication and transport personnel) similarly showed a significant interaction between fusion with Taiwan and willingness to sacrifice for democracy as a sacred value. *Note*: Y‐axis for (A–C) uses a discrete Likert scale (0–6); for (D), a continuous slider scale (0−100).

Field and online studies in North Africa and Western Europe suggest that although identity fusion and sacred values independently motivate willingness to make costly sacrifices, their interaction maximizes willingness to sacrifice (Figure 2). In one study, we investigated two urban Moroccan neighborhoods (Sidi Moumen in Casablanca and Jemaa Mezuak in Tetuan) with a legacy of youth involvement in militant jihad, including bombings in Morocco and Spain (e.g., the 2004 Madrid train bombing, Europe's deadliest terrorist attack to date) and enlistment into Al‐Qaeda and ISIS [118]. About one‐third of sampled residents scored as devoted actors, whose willingness to sacrifice was maximized through the interaction of an absolute commitment to the strict application of Islamic law (Sharia) as a sacred value and fusion with a kin‐like group of Islamist comrades (Figure 2A) [119]. Similarly, in a representative survey in Gaza after 15 months of all‐out war following the October 7, 2023, Hamas‐led attack on Israel, roughly one‐fifth of the population, mostly Hamas supporters who were also fused with Palestine, showed much the same pattern of interaction and willingness to sacrifice for Sharia as the law of the land (Figure 2B) [120]. In a large sample of Spaniards, just 12% tested as devoted actors who considered democracy sacred, and only after being primed with terrorist threats (Figure 2C) [121]. By contrast, in a 2025 island‐wide survey, we found 49% of key Taiwan personnel apt to directly respond to an invasion, identifying as devoted actors—fused with Taiwan and holding democracy as a sacred value—showing a similar interaction effect (Figure 2D).

**FIGURE 3:**
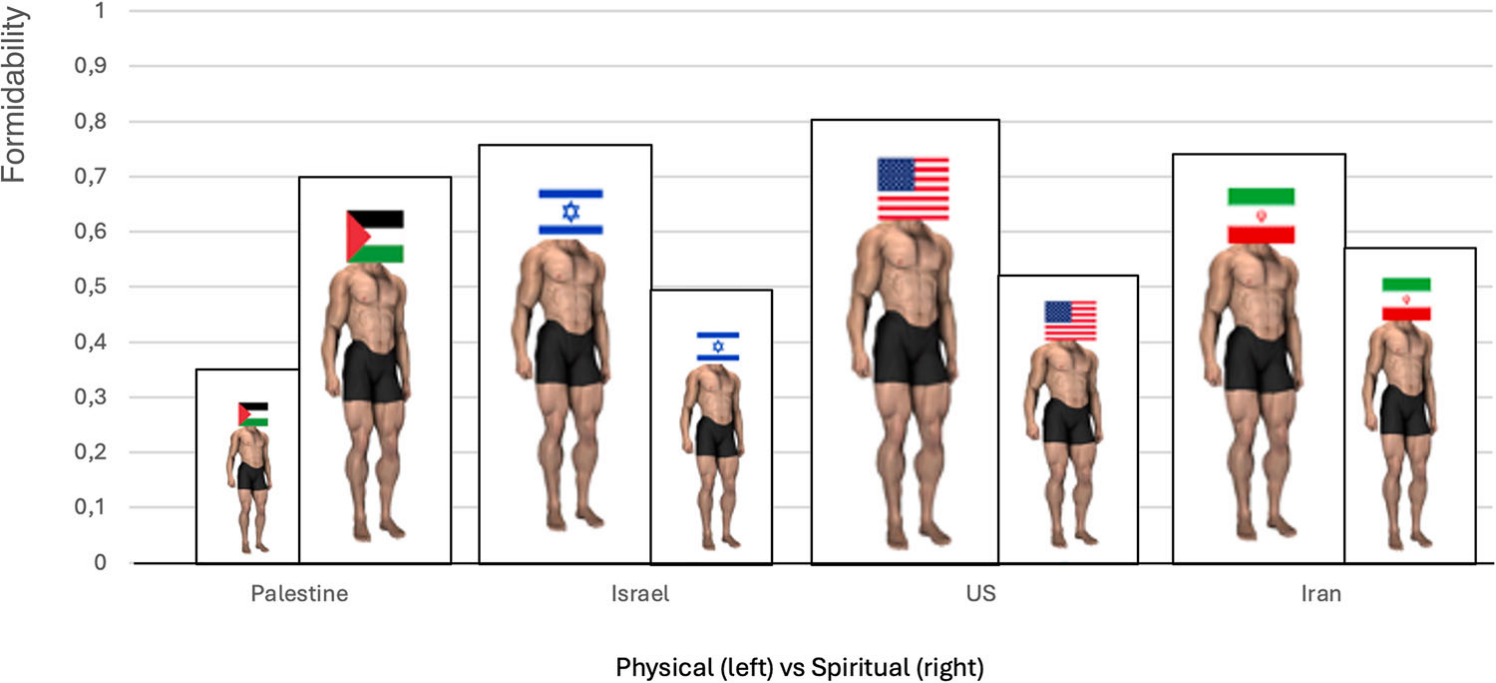
Mean rating of physical versus spiritual formidability of four national groups as judged by Gazans.

From 2015, when the Islamic State was at its maximum extension in Iraq, to 2018, shortly after the fall of Mosul, we carried out field studies with various combatant and support groups (ISIS, PKK, Peshmerga, Sunni Milita, Iraqi Army). We found that willingness to fight and die was greatest for those who: fused with their comrades in arms, committed themselves to uphold sacred values, and assessed “spiritual formidability”—be it their own group, allies, or enemies—as more critical than physical formidability (interpreted in terms of firepower and manpower). The original measure of (physical) formidability was based on the evolutionary principle regarding perception of body size and muscle power as a signal to fight or flee (or in the case of some primates, to accommodate) [122]. To assess formidability, respondents are shown a pair of semi‐nude bodies side by side with a group emblem, such as a flag or banner, attached to their heads. The bodies can be increased or decreased in size and musculature with a slider (Figure 3).

We first discovered the importance of spiritual formidability when ISIS fighters, as well as Kurdish militants of the Marxist‐Leninist PKK, dismissed the very idea of physical formidability as a motivating factor; they literally threw down their tablets and declared that material strength is irrelevant and that only “spiritual strength” (*ruhi bi ghiyrat*, in both Arabic and Kurdish) was important [61]. Fighters recurrently described this as “spirituality with bravery” to defend what is most cherished, “what is in our heart” and “strength of belief in what we are fighting for.” Only the secular PKK fighters matched religious ISIS fighters for willingness to sacrifice for their cause (validated in terms of casualties, time at the front, and so forth), including readiness to abandon comrades deemed willing to compromise their beliefs and, if necessary, sacrifice family for the greater cause of an Islamic Caliphate or the preservation of “Kurdeity” [11]. [Note: The United States and other Western countries consider ISIS and PKK to be terrorist organizations, which may foster resistance in learning proactive lessons from them.]

In our 2025 Gaza survey, we asked participants to compare themselves with Israelis, Americans, and Iranians. The respondents considered Palestinians to be far stronger spiritually than they are physically. This was the opposite of how they perceived Israel, the United States, and even their own putative ally Iran, which they viewed as much stronger physically than spiritually (Figure 3) [120].

More broadly, we find that groups that perceive themselves as relatively weak physically but strong spiritually tend to be those that are more militant or radicalized and willing to continue fighting, even against a far more powerful foe. They leverage their readiness for self‐sacrifice as an advantage over their adversaries. This was true as well for fighters and supporters of ISIS and the PKK. It is also the case for others who may be just as devoted and willing to self‐sacrifice, as we find for the (much smaller) groups of devoted actors willing to sacrifice for democracy in Spain or for peace between Palestinians and Israelis [99]. Collaborating with the US Air Force, we found in studies in Palestine, Iraq, Morocco, Spain, and among Air Force cadets that perception of personal spiritual formidability is more strongly associated with willingness to sacrifice than physical formidability, with this effect mediated by a stronger group loyalty [123].

Not only are devoted actors inspired to action in ways dissociated from material costs and consequences. They can also have an outsize effect on an entire population's WTF. Thus, our Gaza survey indicates that diminished support for Hamas leadership may obscure a larger reality about the role the group plays in the war in Gaza. A majority of Gaza's population continued to be committed to Hamas's ideals: passing the midpoint on an identity‐fusion scale for national sovereignty, Sharia as the law of the land, and “right of return” of refugees and their descendants to homes lost since Israel's creation. In early 2025, Gazans’ view of Hamas was reminiscent of what we found in camps for displaced persons in Iraq soon after the defeat of the Islamic State in Mosul [124]. In those camps, most Sunni Arabs had initially welcomed ISIS as the “revolution” (*thawra*), but judged ISIS rule as more brutal, corrupt, and hypocritical over time. Nevertheless, they remained committed to ISIS's ideals of Sharia rule in a transnational Caliphate, thoroughly rejecting democracy and a unified Iraq as a tyranny of the Shia majority imposed on them by the United States and Iran. Today, ISIS survives in the region, and thrives in the shadows, because it is still able to enlist such people.

## 9 Further Behavioral and Brain Studies Within DAF

**FIGURE 4:**
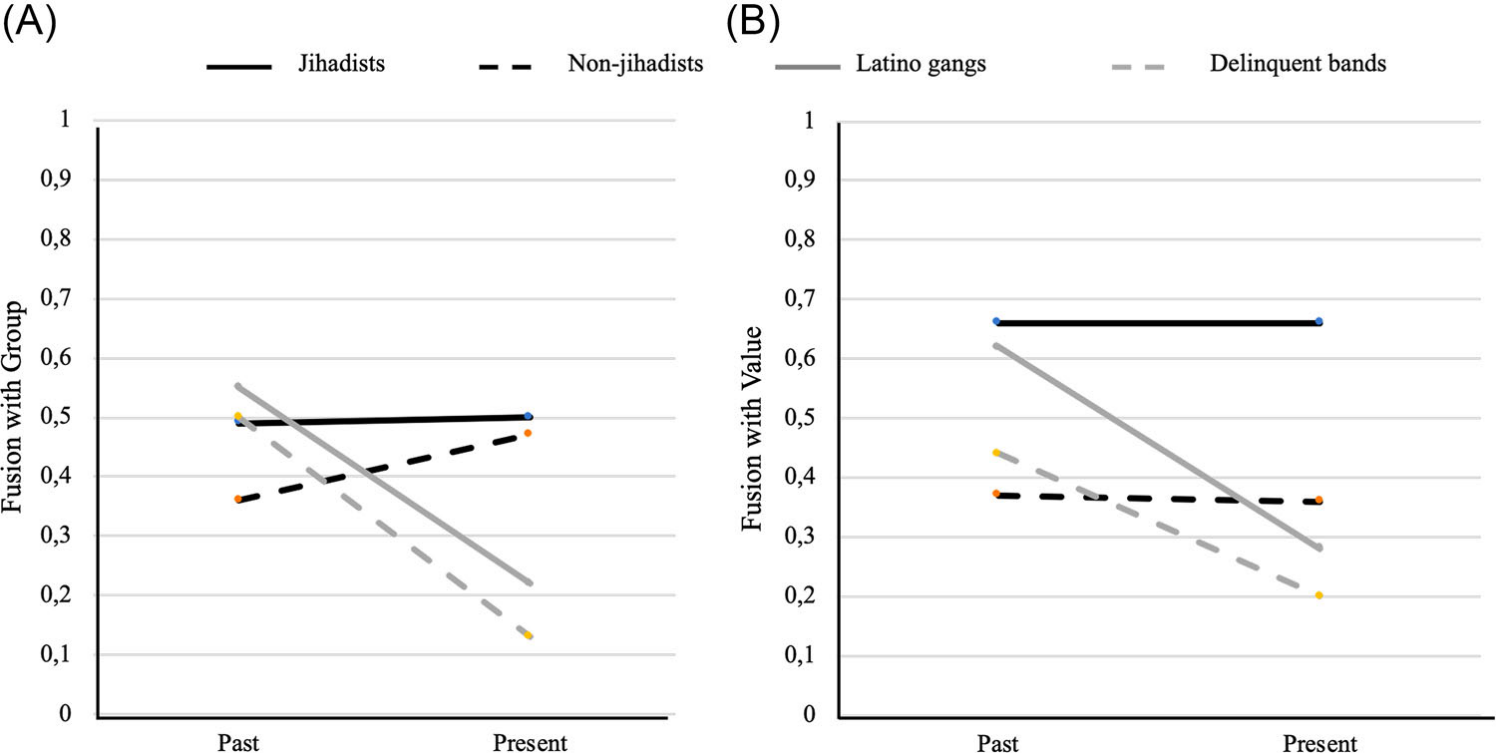
Spain's prisoners’ perceived level of fusion with primary reference group (A) and value (B) over time (time of sentencing to present, average 56.28 months). Jihadists maintain, and non‐jihadist Muslims increase, fusion with primary group (i.e., Muslims), whereas Latino gangs and delinquent bands decrease fusion with their primary criminal groups over time. Jihadist and non‐jihadists maintain fusion with their primary value (i.e., religion), but jihadists show significantly higher levels than non‐jihadists, while gangs and bands show less fusion with value (i.e., honor) over time. Although both jihadists and violent gang members were separated in prison, only jihadists maintained commitment to their group and value.

In a study of convicted jihadists, violent Latino gang members (e.g., MS‐13), and Muslim and non‐Muslim ordinary criminals in 35 Spanish prisons, we found that jihadists, unlike other inmates, remained fused with their group and value over prison time (Figure 4) [115]. Based on findings in other and ongoing studies at the time [114, 115], we used fusion with values as a proxy for sacred values.

**FIGURE 5:**
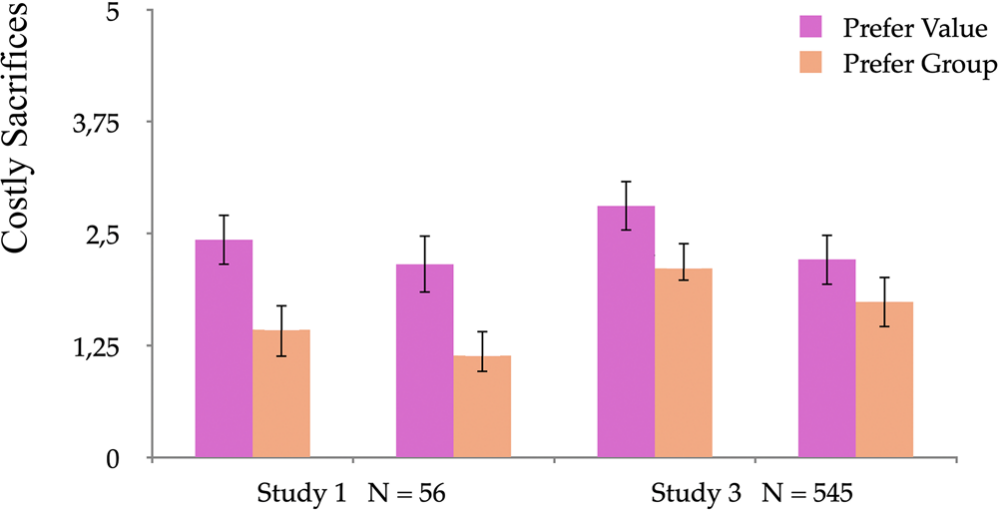
Willingness to make costly sacrifices among participants on the ISIS frontline in Iraq (A) and online in Spain (B) if they judged their families and their other fused groups liable to forsake their sacred values. The scale of costly sacrifices for the frontline field study in Iraq was from 0 to 5; the scale for the online study in Spain was from 0 to 6.

Jihadist prisoners also sacrifice more than other inmates for group and value. But here value takes primacy: shared value is a key enabling factor for fusion with group, and Jihadists show more sacrifices for value than for group [115]. These findings closely track results with frontline combatants in Iraq and online participants in Spain, most ready to self‐sacrifice for their sacred values even more than for their families or other fused groups (Figure 5) [61]. In a forced choice where the group no longer held a value, the most devoted actors opted to reject the group and retain the value.

In parallel, our research group's neuroimaging studies probed willingness to make costly sacrifices among Moroccan immigrants in Spain who professed support for armed jihad and strict conformity with Sharia, and among supporters of Lashkar‐e‐Taiba, a Pakistani associate of Al‐Qaeda. Participants indicated greater readiness to sacrifice against violation of sacred values (e.g., caricatures of Prophet Mohammed) than nonsacred values (e.g., women refusing the veil), with neuroimaging during processing of sacred values revealing inhibition of activity in brain areas associated with cost−benefit and deliberative reasoning [63] but enhanced activity in areas linked to subjective value [125] and rule‐bound judgments (“do it because it's right,” whatever the cost) [113].

Moreover, perception of social exclusion resulted in sacralization of hitherto important but nonsacred values and increased willingness to sacrifice [62]. This converges with research showing that fused individuals who feel excluded become more willing to fight and die [103]; and this result complements our findings from Iran that material disincentives to abandon the nation's nuclear energy program (international sanctions, a version of political exclusion) only increase support among some elements of the population (mostly rural, religious supporters of the hardline regime) for nuclear development as a sacred mission linked to national sovereignty and religion [78, 126]. Motivated by these parallel findings at individual and national levels, a recent study shows significant similarities between behavioral and neural patterns associated with personal and group grievance and perceived discrimination [127].

Further neuroimaging and behavioral studies indicate that far‐right supporters in the United States and Europe are also cued to core cultural values. For example, in social media, they more readily share misinformation about sacred values (e.g., immigrant threats to cultural purity), independent of familiarity, attitude strength, or salience of the issue. Their responses during brain imaging activated a neural network associated with identity processes. Far‐right partisans also disregarded fact‐checking and accuracy reports [128], suggesting that appeals to logical argument, empirical evidence, and familiar forms of scientific, legal, and scholarly validation are largely irrelevant to persuading or changing the minds of true believers.

In brief, the studies presented above indicate that identity fusion, commitment to core cultural values held to be sacred, and spiritual formidability are interacting multipliers and predictors of willingness to make costly sacrifices, including fighting and dying. In other studies involving nearly 12,000 participants from countries in the Middle East, North Africa, North America, and Europe (including Ukraine before and after Russia's 2022 invasion), through mediation analyses, we modeled empirically tested causal relationships between these factors, along with the additional factor of trust [69].

As with spiritual formidability, trust was not a prior concept of our initial research design. Rather, the notion of trust as a driver of WTF, and of distrust as a brake on WTF, spontaneously emerged in field interviews and debriefings with various combatant groups on the ISIS frontline, in studies with imprisoned jihadists and Syrian refugees in Spain, and with displaced persons in Iraq. In our field studies, trust was expressed in somewhat different ways: (1) with reference to groups, individuals, or values (e.g., PKK fighter:  “I trust our leader, Abdullah Öcalan,” “I trust in [the value of] Kurdeity”); (2) in a comparative context (e.g., Peshmerga fighter: “I trust in Kurdistan, not in Iraq because the Iraqi army collapsed and ran from ISIS without a fight”); or (3) as something that could compel individuals leave or reject a group, individual, or value (ISIS fighter: “If the *mujahedin *[holy warriors] were to reject or compromise on *Sharia *[Islamic law], I would no longer trust them”). In each case, trust expresses belief in the reliability and commitment of the group, individual, or value who is trusted, and in the worth and truth of what the trusted group, individual, or value stands for. Trust in the group, leader, or value was measured using up to four reliably covariant items.

**FIGURE 6:**
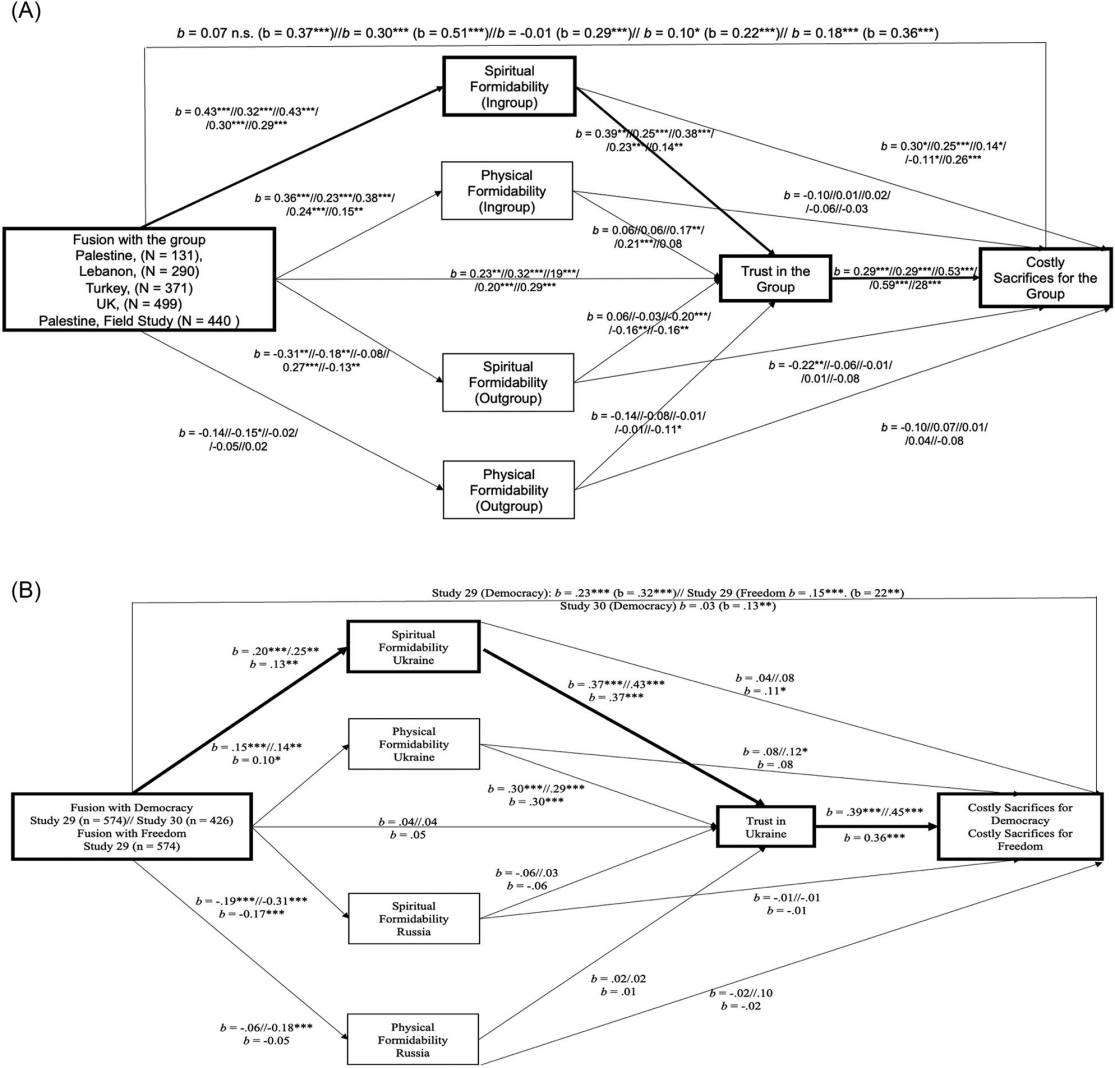
(A) Fusion with the group is positively associated with costly sacrifices via perceived spiritual formidability and trust in five countries. (B) Fusion with the values of Democracy and Freedom is associated with costly sacrifice for these values in Ukraine via perceptions of Ukraine's Spiritual Formidability and Trust in Ukraine.

Here, illustrated in Figure 6 [69], is one validated causal (mediation) model of costly sacrifices, including WTF, in five countries based on empirical data using our measures (Figure 6A), and replicated in 17 studies:

The mediation model Fusion → Spiritual Formidability → Trust → Costly Sacrifices replicates whether fusion concerns a group or a cherished value, such as Democracy or Freedom (Figure 6B, where, again, fusion with value is a sacred‐value proxy).

## 10 Discussion: Scope and Limits of the DAF

The DAF primarily seeks to account for the behavior of individuals engaged in conflict—soldiers, insurgents, militants, active supporters—who are willing to make costly sacrifices for sacred values and fused identities. It focuses on how psychosocial commitment to non‐negotiable values, combined with identity fusion, drives extreme self‐sacrifice beyond rational cost−benefit calculations. While national mobilization efforts and policymaker rhetoric may influence the formation of devoted actors, these are considered enabling conditions rather than central to the framework. The framework does not directly seek to model elite or strategic decision‐making. Although leaders, too, can be devoted actors who are fused with their group and its sacred values, in the studies presented above, leadership is treated as an independent variable, along with other independent variables, such as nationalist or religious framing, existential threats, group hardship or trauma, and ritual and socialization practices that underscore identity and values. These independent variables may also be considered “constructivist” aspects of DAF that contribute to how values, identities, and meaning systems are produced and sustained through historical developments and cultural narratives.

The causal elements of DAF lie in its identification of specific psychosocial mechanisms—such as sacred values and identity fusion—that reliably predict willingness to fight and die, even against overwhelming odds. These mechanisms operate independently of rational cost−benefit calculations, making behavior noninstrumental yet causally explicable. Empirical studies, including brain imaging and field experiments, have shown that when individuals perceive a cause as sacred and feel fused with a group, they are more likely to make extreme personal sacrifices. In addition to sacred values and identity fusion, other mediating variables can systematically intervene in the causal chains linking identity fusion and sacred values to WTF. Examples discussed include spiritual (vs. physical) formidability and trust (in comrades, community, and leaders). The measurement of these mediating variables allows DAF to repeatedly generate testable predictions about combat motivation and group resilience in conflict. In brief, although DAF is a hybrid of both constructivist and causal elements, it offers causal explanations of behavior—in particular, WTF—through testable mechanisms, while drawing constructivist insight into how sacred values and fused identities are produced and sustained.

When considering both casual and constructivist elements, we can see what both enable and prevent people from becoming devoted actors. Soldiers, for example, become devoted actors when they internalize sacred values, feel fused with their comrades, nation, or movement, and perceive a conflict as a cause worth dying for. These conditions often arise in contexts of existential threat, moral clarity, charismatic leadership, and intense group bonding through ritual and hardship. Under some conditions, devoted actors identify with small family‐like groups of “brothers in arms,” as with certain Libyan militia groups [101]. In other circumstances, fighters may be willing to sacrifice even their families for the sake of a greater collective identity and cause, as with Kurdish PKK and Peshmerga fighting for “Kurdeity” and ISIS *inghimasi* (“penetratrors,” suicide attack troops) fighting for an Islamic Caliphate [129].

People fail to become devoted actors when they do not perceive a sacred cause worth dying for, do not feel deeply bonded to a group, or live in environments that suppress shared moral narratives. Without these conditions, behavior remains pragmatic, calculated, and conditional—rather than absolute and sacrificial. Take the Vietnam War: For sociologist Charles Moskos, who served in Vietnam and studied its combat veterans, American motivation was based on both a more personal and individualistic perspective and a broader social vision grounded in universal rather than nationalistic values. Moskos observed that American soldiers often focused on individual survival rather than on instituting democratic ideals. A key factor influencing this mindset was the rotation system, where each soldier had a predetermined tour length, leading many to concentrate on enduring their service until their return date: “The overriding importance of the rotation system as a determinant of combat motivation and the corresponding likelihood for soldiers to see the war in very private and individualistic terms” [130].

By contrast, the Viet Cong and North Vietnamese were apparently more driven by strong nationalist and moral beliefs of equal and fair treatment (colored but not determined by communist ideology). Their cause and comradery were long forged in fire against a brutal Japanese occupation and the French Indochina War, and under a largely noncorrupt and cohesive leadership structure characterized by dedication and effectiveness. American soldiers described the selfless bravery and sacrifice of the North Vietnamese and Viet Cong because “they believed in something” and “knew what they were fighting for” [131]. Table 1 provides further examples of how identity fusion and sacred values, along with other contributing factors, enable or prevent devoted‐actor commitment.

**TABLE 1:** Conditions enabling versus preventing devoted actor commitment (with examples).

Factor	Enables devotion	Example of enabling	Prevents devotion	Example of preventing
Sacred values	Values seen as non‐negotiable and morally absolute	Viet Cong fighters saw national liberation as sacred	Values seen as instrumental or negotiable	US soldiers in Vietnam often lacked sacred framing; many waited for rotation home
Identity fusion	Strong emotional fusion with group; group's fate = personal fate	Kurdish PKK fighters fused personal and communal liberation	Weak identification with group; personal over collective identity	Fragmented units in Iraqi Army versus ISIS, 2014 collapse
Existential threat	Clear threat to group survival or identity	Ukrainian soldiers resisting Russian invasion post‐2022	No immediate or existential threat	Western European and Taiwanese societies (until recently) lacked urgency despite long‐standing threat
Cohesive moral narrative	Shared story of just struggle and enemy wrongdoing	Thermopylae (Spartans) Identity fusion with *polis*, “a partnership of citizens in a good life” (Aristotle, *Politics*); sacred duty and honor as embodiment of divine order and ancestral law (*nomos*)	Conflicting or unclear moral narrative	Lebanon struggles to create national narrative amid ethnic fragmentation
Socialization and ritual	Shared tribal customs, religious symbols and ceremonies, martyr veneration	Taliban use of religious and tribal traditions and rituals	Lack of shared meaning or unifying experiences	Heterogenous Afghan National Army units with low cohesion and morale
Trusted moral leadership	Charismatic or morally credible leaders inspire belief in cause	Ho Chi Minh and Vo Nguyen Giap unified Vietnamese cause across classes and regions	Distrust in corrupt or illegitimate leadership	US‐backed governments in Vietnam, Iraq, or Afghanistan often viewed as corrupt
Collective experience of hardship	Shared suffering builds solidarity and mutual sacrifice	WWII Soviet defenders of Stalingrad experienced shared siege trauma	Individual comfort, fragmented experiences weaken group bonds	Some Western societies with strong individualist cultures during peacetime

Aspects of DAF overlap with both primary group theory and cohesion theory in the literature on combat motivation. But all three differ in focus and intensity. Primary group theory relates to close‐knit, emotionally intimate family‐like groups, where strong interpersonal bonds drive loyalty and behavior. For example:

The primary group was the core of the German soldier's morale. The small combat group, the squad, the section, and the platoon, was the immediate social world …. His sense of obligation, of duty, and of loyalty was, above all, directed toward the members of this group. This group was, in turn, the source of his self‐respect and the measure of his standard of performance. [132, 133]

Other authors also note this sense of group empowerment among German soldiers “strong enough in the group's later stages to approximate group immortality and the will to fight on against desperate odds” [134].

Within DAF, however, identity fusion goes further, describing a deep, visceral sense of oneness with a group where personal and group identities are merged with the group's sacred values. This merging—whether for family‐like groups of close friends or comrades or whole nations and movements—can lead to extreme self‐sacrifice for the group and belief that the group itself is ultimately invincible and will be triumphant, even if only its values survive: “Only a belief in Nazism could explain why the Wehrmacht continued to be so aggressive and determined on the offensive, and so dogged and tenacious on the defence, despite often very high numbers of dead and wounded” [135]. The interaction of identity fusion and sacred values was evident among units of Germany's Waffen‐SS, the ideologically driven military wing of the Nazi Party's elite SS (*Schutzstaffel*) paramilitary organization. Throughout the war, it played a central role in frontline combat and atrocities (much like the ISIS *inghimasi* in recent years), suffering casualty rates far beyond those of the Allies [136] and impervious to the roughly one‐third degradation that usually leads to unit entropy and collapse [137, 138]. Thus: “General [Johannes] Friessner [commanding German regular forces] can count for defense of the [Hungarian] capital on SS units that operate under the name Death Volunteers” [139].

Jasen Castillo's cohesion theory broadens the perspective of primary group theory, arguing that soldiers fight not only for each other but also for belief in a cause or national identity [140]. In addition to group bonds and ideological commitment, cohesion theory also integrates institutional factors, by identifying multiple forms of cohesion—social (shared identity and friendship), task (commitment to shared mission), ideological (like nationalism or religion), and vertical (e.g., between officers and enlisted troops)—as key determinants of unit effectiveness under fire. For example, when a regime exercises tight societal control and grants its military significant autonomy in training, it creates what Castillo terms “messianic” militaries. These forces are highly cohesive, motivated by a strong national or ideological mission—even in the face of apparent defeat, as with Nazi Germany in WWII [133] and North Vietnam in the Vietnam War [141]. By contrast, Democracies or less‐controlled regimes that allow strong military autonomy, while not as fast to mobilize resources, can adapt to changing circumstances and excel tactically—until the war becomes unpopular or unwinnable. While both cohesion theory and DAF recognize the role of group loyalty and ideology or values, the devoted actor is rooted in psychological commitment to transcendent causes that drive individuals to extreme sacrifices in ways dissociated from group survival, material incentives, institutional support, or formal cohesion.

Messianic militaries do share fused and transcendent elements of DAF [140]; however, less‐controlled democratic regimes also can. R.V. Jones, head of Britain's WWII Air Ministry's “scientific intelligence” effort, expressed such devotion as follows:

I used to look at my wall map every morning and wonder how we could possibly survive. Anyone in his right sense would do the best deal he could with Hitler—but we had no thought of it… there was that white glow overpowering, sublime that ran through our island from end to end…. It must lie very deep down among human emotions… at facing dangers in which he may easily perish as an individual but also a subconscious knowledge that any society which has a high enough proportion of similar individuals is all the more likely to survive because of their sacrifice. [142]

Analyzing Soviet (Russian) resistance in WWII, Richard Overy captures a similar fighting spirit, a will to ensure the persistence of ontological significance, of group identity and values, no matter the cost:

There have been ample opportunities since 1945 to show that material superiority in war is not enough if the will to fight is lacking… The Second World War was … popularly perceived to be about issues of life and death of whole communities…. They were issues… ‘worth dying for’. [143]

There is perhaps an evolutionary logic to such commitment: to what Darwin deemed “highly esteemed, even sacred” spiritual and moral virtues that “give an immense advantage” to one group over another when possessed by devoted actors who “by their example excite… in a high degree the spirit” in others to sacrifice for cause and comrades [144]. DAF is not a replacement for realism or moralism. It does not ignore the importance of rational decision‐making, plays for power, or material incentives in initiating, sustaining, or ending group conflicts. But DAF can offer added insight into group conflict based on different assumptions about human motivation, the nature of values, and the logic of decision‐making in conflict. Realism focuses on instrumental interests and strategic interaction between states that weigh the costs and benefits of waging war under conditions of anarchy. By contrast, DAF focuses on moral conviction and identity in accounting for how individuals and groups become willing to fight and die for causes that are highly resistant to bargaining or deterrence.

DAF also is not a substitute for moralism, but it differs in focus and explanatory function. While moralism and DAF both emphasize values and beliefs, DAF does not endeavor to ascertain whether a conflict or conduct in war is ethically justified. It is not normative or prescriptive in seeking to evaluate whether behavior is right or wrong. Rather, DAF describes relations between variables that interact to provide causal explanations of how people in conflict come to make costly sacrifices, including fighting and dying.

To be sure, few conflicts rise to the levels of extreme sacrifice associated with Thermopylae, Masada and the Alamo, or the Battles of Britain, Stalingrad and Dien Bien Phu—conflicts that DAF is ideally poised to assess along with other ongoing and seemingly intractable conflicts (Israel−Palestine, Russia−Ukraine). But even for less extreme cases, DAF can help clarify causal factors, such as identity fusion and sacred values, that may be present but less visible. It may also help reveal the limits of rationality (that go beyond the “bounded rationality” and “biases and heuristics” that stem from lack of information or cultural awareness, memory limits, and other cognitive limitations, or time constraints) [145]. In illuminating the importance of values and identity, even low‐intensity settings (e.g., in defending a village or inspiring activism) may be better explained and understood.

## 11 Coda: How Ontological Significance Impacts the Israel−Palestine Conflict

Don't ask me what the political solution is to be. . . . Our sacred duty is to fight, to resist occupation of our sacred land and change the conditions of our people. That is our duty, our sacred duty. . . . I will not accept the existence of Israel. I will never accept the existence of a state of Israel. Never, ever.

Ramadan Shallah (interviewed by S. Atran and R. Axelrod), General Secretary, Palestinian Islamic Jihad, Damascus, December, 2009 [146]

There is no such thing as a Palestinian nation. There is no Palestinian history. There is no Palestinian language.

Israel Finance Minister Bezalel Smotrich, speaking in Paris, March, 2024 [147]

The overall take from our behavioral and brain studies presented within the DAF is that WTF among the most committed violent extremists and warriors in lethal conflicts may remain imponderable—and associated security challenges seemingly insoluble—if we observe matters exclusively through the lens of instrumental rationality; however, much utilitarian calculations may also be involved in the competition for power and resources within and between groups. Such findings indicate that deradicalization programs focused primarily on buying off would‐be extremists and devoted adversaries with material incentives or punishments could well backfire, and business‐like negotiations favored in diplomatic approaches to intractable conflicts would likely fail.

Consider in this respect a previous series of field studies in the Israel−Palestine theater. My colleagues and I surveyed more than 5000 Palestinians and Israelis from 2004 to 2013, questioning across the political spectrum, including refugees, Hamas supporters, and Israeli settlers in the West Bank and Gaza [12, 77]. We also posed similar questions to Israeli and Hamas leaders [76, 148]. We asked them to react to hypothetical but realistic compromises in which their side would be required to give away something of ontological significance that it valued in return for lasting peace. Participants were asked to imagine (and confirm their belief in the credibility) that the United States (or the UN Security Council or the European Union) would commit to the peace deal. Each population survey used a between‐subjects design: one representative sample would be given a straight‐up offer in which each side would make difficult concessions in exchange for peace (taboo condition); and a second sample would be given a scenario in which their side was granted an additional material incentive (taboo+ condition).

For example: Suppose the UN Security Council organized a peace treaty between Israel and the Palestinians where Palestinians would give up their right to return to their homes in Israel, and there would be two states, a Jewish state and a Palestinian state in the West Bank and Gaza. Second, in return, the United States and EU would give the Palestinian state $10 billion a year for 100 years (in other versions, each Palestinian would receive compensation).

Across the political spectrum, an overwhelming majority rejected the initial solutions offered. For many, this was because the values involved were sacred to them. Nearly, half the Israeli settlers surveyed refused to trade any land in the West Bank—territory they believe was granted to them by God—in exchange for peace. Just before Jewish settlers were evacuated from Gaza, we presented a compensation bundle that Israel's government was then actually offering, but which they fiercely rejected. A majority of Palestinians viewed sovereignty over Jerusalem in the same light, and more than four‐fifths felt the right of return was sacred.

**FIGURE 7:**
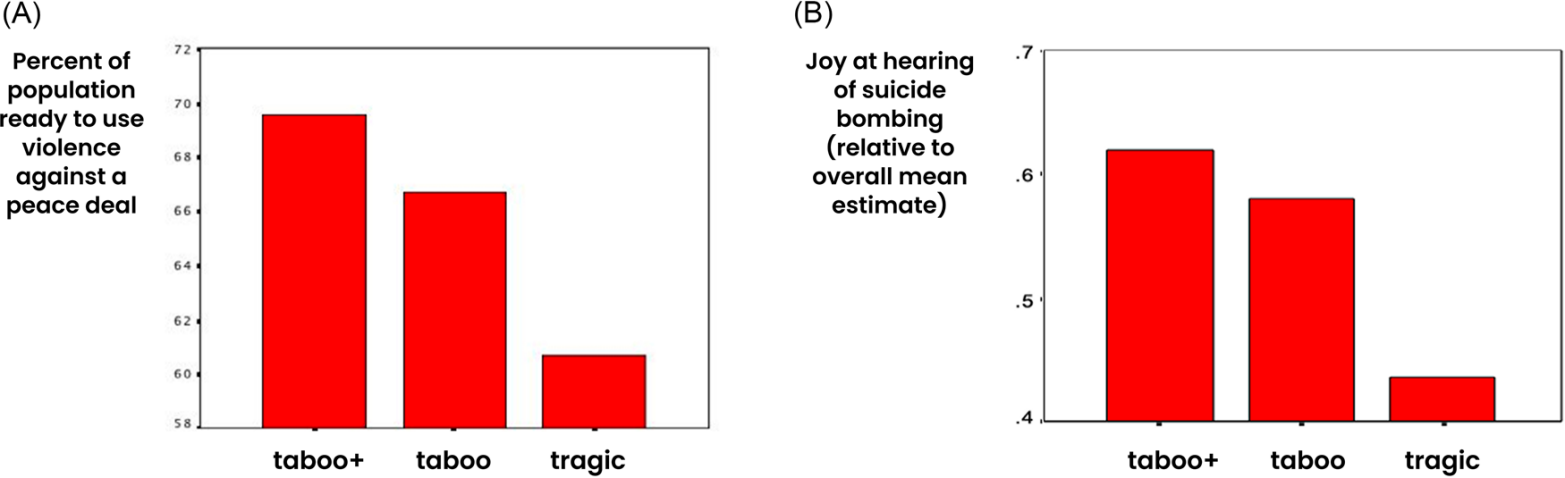
Palestinian refugees’ responses to trading off the group value of “Right of Return” for each of the three conditions for peace: taboo (offer to abandon “Right of Return” for permanent peace), taboo+ (added material incentive to the taboo offer), tragic (Israeli apology as a symbolic concession to the value, without a material offer, in exchange for peace). (A) Refugee predictions of percent of Palestinian population ready to use violence to oppose each of the three peace deals: linear trend. (B) Refugees reporting “joy” a hearing of suicide bombing relative to overall mean estimate (i.e., where the average joy level across all three conditions is set as the baseline).

As for sweetening the pot, the greater the monetary incentive involved in the deal, the greater the disgust, as well as increased support for further violence in pursuit of the conflict. Indeed, actual offers of material compensation to Jewish settlers to leave Gaza and abandon the right to settle in “Greater Israel” (*Eretz Yisrael Ha‐Shleimah*) resulted in increased readiness to use violence against their own government, whereas Palestinian expressions of support and joy for suicide attacks ramped up significantly (Figure 7) [77]. From realist and rational bargaining perspectives, these clearly expressed “irrational” preferences.

Fortunately, our work also offers hints of another, more optimistic course. Absolutists who violently rejected offers of money or peace for sacred land were considerably more inclined to accept deals that involved their enemies making symbolic but difficult gestures (tragic tradeoff condition). For example, Palestinian hardliners, including Hamas leaders who were presented the same set of tradeoffs (but necessarily in a within‐subjects design), were more willing to consider recognizing Israel's right to exist if Israelis offered a public apology for Palestinian suffering in the 1948 war.

In Cairo, Mousa Abu Marzouk (then‐deputy chairman of Hamas) said “no” to any tradeoff for peace without granting a right of return. He became angry when the idea of substantial American aid for rebuilding was added: “We do not sell ourselves for any amount.” But when offered a potential Israeli apology for 1948, he conceded: “Yes, an apology is important, as a beginning. It's not enough because our houses and land were taken from us and something has to be done about that.” Similar responses came from Hamas's top official in Gaza, Ismail Haniyeh, and in Damascus from then‐chairman Khaled Meshaal. They indicated that even the right of return, though held to be sacred, can be reframed so as to remain non‐negotiable in principle but allow practical accommodation if accompanied by sincere and meaningful symbolic gestures, such as an Israeli apology for what happened to the refugees, acceptance of a token return of refugees and their descendants, and “blood payment” (*diya*) of financial compensation to victims or heirs of a victim killed, physically harmed or for loss or damage to property. In statements to me for public attribution in the *New York Times* [148], they indicated that peace (*salaam*) may be possible, and not merely a time‐limited truce (*hudna*), upon sincere recognition of the historical harm done to Palestinians, particularly the refugees. This would have to be followed by guarantees of “a sovereign Palestinian state over all our lands within the 1967 borders” credibly enforced by a “balance of arms and power” [70]. These offers raised objections by others committed to the elimination of Israel through war (*harb*), such as the leaderships of Hamas's Qassam Brigades and of Palestinian Islamic Jihad [20].

As for Israeli leaders, they responded that they could live with the partition of Jerusalem and borders very close to those existing before the 1967 war if Hamas and other major Palestinian groups sincerely respected Israel's right to exist. Binyamin Netanyahu told us in Jerusalem: “O.K., but the Palestinians would have to show that they sincerely mean it, change their textbooks and anti‐Semitic characterizations” [70, 76, 149]. Of course, talk of peace, especially among leaders, could represent posturing, and may well have changed since. However, previous research indicates that regional leaders’ commitments to sacred values track popular sentiment. Leadership decisions to resort to violence have tended to follow rather than precede public support for violence. With Hamas, this time‐lag pattern has been consistent, based on more than two decades of polling by the Palestinian Center for Policy and Survey Research [150].

**FIGURE 8:**
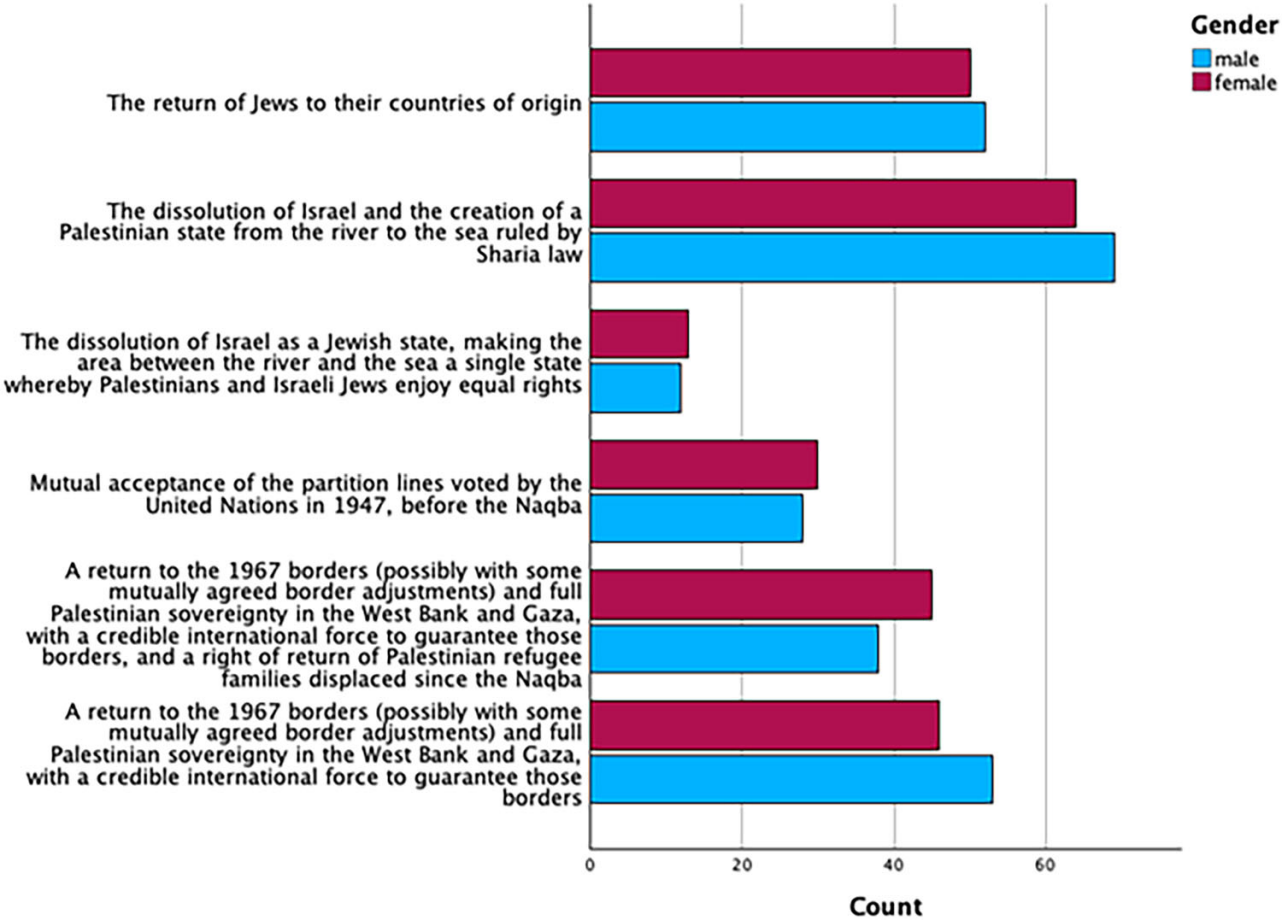
Gazans’ responses to the question: To end the Palestinian−Israeli conflict, which of the following scenarios do you think is a realistic solution that the Palestinian people should accept? January 2025 representative field survey of 252 men, 248 women (margin of error ± 4%). Note that the end‐state solution preferred by many Western supporters of the Palestinian cause is by far the solution least acceptable as realistic by Gazans themselves.

Granted, when leaders of Hamas and their principal ally, Palestinian Islamic Jihad, make such statements, they also may be engaging in insincere posturing aimed at relieving Israeli military pressure, as Israeli leaders have claimed. Still, there is evident willingness among Gaza's population even after many months of catastrophic war to countenance an outcome to the Palestinian−Israeli conflict that falls short of the elimination of Israel, which the majority of Gazans consider to be the most acceptable and realistic set of solutions (Figure 8); however, certain material as well as symbolic concessions by Israel would be needed, as with matters of “Balance of Power” and “Right of Return.”

Balance of Power, a negotiable material good, would ensure physical security. Right of Return, a non‐negotiable but reinterpretable sacred value, would provide a measure of ontological significance and security, that is, of having a place in the world: a respectful recognition of what Palestinians often cite as the conflict's central issue, *al‐‘Ard hiya al‐Ard*, “Land is [family and community] Honor.” To cede the land for conversion into a tourism and technology hub, as US President Donald Trump had once pressed Gazans to do [151]—echoing the Israeli far‐right's longstanding call for population “transfer” (*ha'avarah* in Hebrew)—would mean ceasing to be Palestinian, to exist in their own right. It likely would only inflame the simmering wound of generations of displaced Palestinians, unassimilated through a combination of intentional refusal and the refusal of host nations to accept them, yearning to return to the land as diaspora Jews once longed for Zion.

Regarding the recent war in Gaza, Harvard's Stephen Walt contends in a realist vein that: “framing this conflict in moral terms makes it harder to reach a peace settlement, because anything short of total victory inevitably invites a powerful backlash from critics fearing that these critical values are being sacrificed” [3]. Rather than seek ways to accommodate clashing values—for example, through sincere symbolic concessions or creative reframing—he contends the sides should avoid dealing altogether with “indivisible” moral disputes and focus instead on bargaining over divisible goods such as territory, resources, and power.

Like John Mearsheimer [152], Walt condemns Israel's brutal warfare as serving no real military purpose and supports cutting US military aid as a realist path to ending the Gaza war—and to addressing the power imbalance that perpetuates the broader conflict [153]. Yet, the century‐long Israel−Palestine conflict is more than one side's relentless drive for power over the other. Perhaps redressing today's power imbalance can open the way to settling the broader dispute, though not on realist grounds alone. Our studies suggest that directly engaging, rather than sidestepping, each side's seemingly irreconcilable values bound to identity—“who I am, and what we are”—can unlock deal‐making, even on material issues, in otherwise intractable conflicts.

## Conflicts of Interest

The author declares no conflicts of interest.

## Human Ethics Approval Declaration

Human Subjects: All studies and findings presented were previously approved by IRBs of the University of Michigan, University of Oxford, John Jay College of Criminal Justice, Universidad Nacional de Educación a Distancia (UNED Madrid), the Universitat Autònoma de Barcelona, and the US Air Force Office of Scientific Research, US Office of Naval Research, and Artis International, as indicated in the original studies cited in the reference section.

## Data Availability

Data sharing is not applicable to this article as no datasets were generated or analyzed during the current study.
